# Effects of Enteric-Coated Formulation of Sodium Bicarbonate on Bicarbonate Absorption and Gastrointestinal Discomfort

**DOI:** 10.3390/nu16050744

**Published:** 2024-03-05

**Authors:** Fang-Lin Jiang, Dong-Ho Jeong, Seon-Ho Eom, Hae-Moon Lee, Bong-Jin Cha, Ju-Seong Park, RyoonKyoung Kwon, Jeong-Yeon Nam, Hyun-Seon Yu, Su-Hak Heo, Chul-Hyun Kim, Keon-Hyoung Song

**Affiliations:** 1 National Traditional Sports Teaching and Research Section of Hunan Province, College of Physical Education, Hunan Normal University, Changsha 410012, China; 2 Department of Pharmaceutical Engineering, College of Medical Sciences, Soonchunhyang University, Asan 31538, Republic of Korea; 3 R&D Center, Jinyang Pharm. Co., Ltd., Seoul 08826, Republic of Korea; 4 Department of Sports Medicine, College of Natural Sciences, Soonchunhyang University, Asan 31538, Republic of Korea; 5 College of Pharmacy, Seoul National University, Seoul 08826, Republic of Korea; 6 Department of Medicinal Bioscience, Konkuk University, Chungju 27478, Republic of Korea

**Keywords:** sports supplement, enteric-coated tablet, oral administration, pharmacokinetics, drug delivery

## Abstract

Sodium bicarbonate is used as an ergogenic supplement to enhance people’s performances in various exercises. This study aimed to evaluate the effects of intestinal delivery of sodium bicarbonate on bicarbonate absorption and associated side effects in an experimental human trial. After preparing and assessing enteric-coated and uncoated sodium bicarbonate tablet formulations, pharmacokinetic analysis and gastrointestinal symptom tests were performed after oral administration in the human body. The dose required to increase blood bicarbonate concentration over 5 mmol∙L^−1^ for the purpose of improving performance during high-intensity exercise was also determined. Enteric-coated tablet formulation protects sodium bicarbonate under acidic conditions and releases bicarbonate in the intestine. Enteric-coated tablet formulation also reduced the oral dose required to achieve a blood bicarbonate concentration over 5 mmol∙L^−1^ from 300 mg∙kg^−1^ of uncoated tablet formulation to 225 mg∙kg^−1^. Gastrointestinal discomfort was significantly decreased for the group given 225 mg∙kg^−1^ enteric-coated tablets compared to that given 300 mg∙kg^−1^ uncoated tablets. These results suggest that enteric-coated tablet formulation could reduce the oral dose required in order to achieve a blood bicarbonate concentration over 5 mmol∙L^−1^ by 25%, from 300 mg∙kg^−1^ to 225 mg∙kg^−1^, along with its ability to reduce gastrointestinal discomfort associated with the dosage.

## 1. Introduction

Sodium bicarbonate is used to treat metabolic acidosis with low blood pH levels due to reduced concentrations of bicarbonate ions in the plasma [1,2,3]. It is assumed to contribute to acid–base balance by buffering excess hydrogen ions [3]. This therapeutic mechanism could be applied to acidic metabolism in skeletal muscles during high-intensity exercise. High-intensity exercise can change the intramuscular metabolic profile, which includes the accumulation of excess hydrogen ions, one of the factors contributing to reduced exercise performance [4,5]. The accumulation of hydrogen ions can cause acidification in the muscle, which is associated with muscle fatigue and muscle contraction disturbances [6,7,8]. Thus, sodium bicarbonate has been employed as an ergogenic supplement in an attempt to increase the extracellular buffering capacity and increase the rate of removal of accumulated hydrogen ions from muscles, ultimately delaying the onset of muscle fatigue and improving exercise capacity during high-intensity exercise such as cycling, rowing, and sprinting [9,10,11,12].

Over the past few decades, many papers have shown that sodium bicarbonate as an ergogenic supplement can enhance people’s performance in various exercises, including running, cycling, rowing, sprinting, and swimming [13,14,15,16,17,18,19,20,21]. The International Olympic Committee (IOC) consensus has stated that sodium bicarbonate is one of the supplements with direct performance benefits, with good to strong evidence. It enhances performance (~2%) in short-term, high-intensity sprints lasting ~60 s in duration for 10 min [22]. 

The common or recommended dose of oral sodium bicarbonate supplement is 300 mg·kg^−1^ body mass [23,24,25,26,27,28], which can increase the concentration of blood bicarbonate ions by approximately 5–6 mmol·L^−1^ [29,30,31]. Such levels are considered sufficient to maintain or improve the buffering and contraction capacity of muscles [29,31]. However, administration of sodium bicarbonate supplements is associated with adverse effects, i.e., gastrointestinal symptoms, in ~30% of athletes [25,26,27]. The most commonly reported side effect is gastrointestinal discomfort, including stomach bloating, belching, bowel urgency, stomachache, stomach cramps, flatulence, diarrhea, nausea, and vomiting [32,33]. Some of these side effects might be caused by carbon dioxide gas, which is produced through carbonic acid after neutralization of sodium bicarbonate by gastric acid in the stomach [34]. Recent studies have suggested bariatric surgery as a gastric bypass model [35], as well as gastro-resistant formulations (i.e., embedded [4,36] and enteric-coated capsules [5,36]) to minimize gastrointestinal side effects for a limited duration of time [5,36]. 

Enteric coating is the usual procedure used to protect orally administered formulations from damage by acidic pH to avoid undesirable effects on the stomach, and is used to increase absorption in the intestine [37]. Bicarbonate ions are known to be transported into the blood through the stomach [38,39]. They are also absorbed by an active ion transport process involving hydrogen ion secretion in the small intestine [39,40]. Given that sodium bicarbonate could become instable due to gastric acid, enteric coating might increase bicarbonate absorption while reducing its side effects [37,38,39,40]. However, a recent study of enteric-coated sodium bicarbonate capsules has reported that blood bicarbonate concentrations and group gastrointestinal symptom scores are not significantly different between groups administered enteric-coated capsules and uncoated gelatin capsules [5]. On the other hand, another paper has reported that blood bicarbonate concentrations were lower in the group administered enteric-coated capsules than in the group administered uncoated gelatin capsules [36]. 

To clarify such a discrepancy, the aim of this study was to evaluate the effects of intestinal delivery of sodium bicarbonate on bicarbonate absorption. After uncoated and enteric-coated tablet formulations were prepared and evaluated, an experimental human trial was conducted by administering 300 mg·kg^−1^ body mass, the typical dose of oral sodium bicarbonate supplements, and 225 mg·kg^−1^ body mass, the dose obtained in preliminary studies.

## 2. Materials and Methods

### 2.1. Materials

Sodium bicarbonate (≥99.9%) was purchased from Ottogi Corp. (Anyang, Republic of Korea). Hydroxypropyl methylcellulose (Vivapharm Hypromellose 2910), triethyl citrate (≥99.0%), and magnesium stearate were obtained from Whawon Pharm. Co., Ltd. (Hwaseong, Republic of Korea), TCI Co., Ltd. (Tokyo, Japan), and Faci Asia Pacific Pte Ltd. (Singapore), respectively. Opadry (04K19229) and Acryl-Eze (93018509) were donated by Colorcon Inc. (Harleysville, PA, USA). All solutions were prepared with high-performance liquid chromatography (HPLC)-grade water purified with Arium Mini Plus (Sartorius, Goettingen, Germany) and cellulose nitrate membrane filters (47 mm, 0.2 μm, Whatman, Maidstone, UK). All other reagents were of analytical grade or better. 

### 2.2. Participants in the Experimental Human Trial 

Eighteen healthy males with a mean age of 23.3 ± 1.7 years, height of 1.75 ± 0.01 m, and body weight of 74.0 ± 3.2 kg (mean ± standard error of the mean) were recruited for this study. These participants had not regularly ingested any buffering agents, supplements, or medications in the previous six months.

They had no gastrointestinal-related disorders. Exclusion criteria were: (1) those with hypertension, (2) those with renal impairment, and (3) those with a sodium-restricted diet. This study was approved by the Institutional Review Board (IRB) on Human Subjects Research and Ethics Committees at Soonchunhyang University (approval No. 1040875-202104-BR-034), in compliance with the Declaration of Helsinki. All participants provided written informed consent to participate in this study after the protocol, study requirements, benefits, and risks had been explained and questions had been answered. 

### 2.3. Preparation of Uncoated and Enteric-Coated Sodium Carbonate Tablets for the Experimental Human Trial

An aqueous solution of HPMC (7% *w*/*v*) was top-sprayed onto sodium bicarbonate powder in a lab-scale fluid bed system (PFB-L, PTK, Gimpo, Republic of Korea) for 40 min at a feeding rate of 30 mL/min, with an inlet temperature of 85 °C and an outlet temperature of 45 °C, to produce sodium bicarbonate granules containing 2% (*w*/*w*) HPMC. Sodium bicarbonate granules continued to be fluidized and dried for 20 min at 45 °C after the HPMC solution had been consumed entirely. After drying, these granules were passed through a USP standard sieve (No. 20) to remove coarse particles. Sieved granules were blended with 0.05% (*w*/*w*) magnesium stearate to provide lubrication, and then compressed into tablets at 1700 psi in a rotary tablet press (GRC-14, Sejong Pharmatech Co., Ltd., Incheon, Republic of Korea) equipped with 9.5 mm round punches. Tablets were further evaluated and administered in the experimental trial as uncoated sodium bicarbonate tablets. 

Uncoated tablets were initially coated with an aqueous solution of Opadry (16% *w*/*v*) to form a seal coat. After seal-coating and subsequent drying, the enteric coating was produced using an aqueous mixture of Acryl-Eze (25% *w*/*v*) and triethyl citrate (2.5% *w*/*v*) for seal-coated tablets. The coating process for both seal-coating and enteric-coating was performed with a tablet coater (SFC-30, Sejong Pharmatech Co., Ltd., Incheon, Republic of Korea) equipped with a peristaltic pump (L/S, Masterflex, Radnor, PA, USA) and a magnetic stirrer (Misung Scientific Co., Ltd., Yangju, Republic of Korea) using an inlet air temperature of 55 °C, an exhaust air temperature of 40 °C, and a pan speed of 8 rpm. 

Uncoated and enteric-coated tablets were evaluated for weight, diameter, thickness, hardness, and friability using a TBH300MD (Erweka, Langen, Germany) and a FAT-10 (Labfine Corp., Gunpo, Republic of Korea). The disintegration time was determined using a USP tablet disintegration test apparatus (DIT-200, Labfine Corp. Republic of Korea) and pH 1.2 and pH 7.4 solutions.

### 2.4. Experimental Human Trial

Participants were required to abstain from food, alcohol, and vigorous exercise for 12 h prior to experiments. Exercising was not allowed during the trial because it could affect bicarbonate absorption. In the experimental trial, the following sodium bicarbonate supplement formulations were administered orally: (1) uncoated tablets equivalent to 300 mg of sodium bicarbonate per kg body mass; (2) uncoated tablets equivalent to 225 mg of sodium bicarbonate per kg body mass; and (3) enteric-coated tablets equivalent to 225 mg of sodium bicarbonate per kg body mass. Each prepared uncoated tablet and enteric-coated tablet contained 432 mg of sodium bicarbonate. This is equivalent to 0.69 tablets per kg of body mass when administering 300 mg of sodium bicarbonate per kg and 0.52 tablets per kg of body mass when administering 225 mg per kg. The number of tablets the participant took was determined by converting the number of tablets according to the administration group and body mass and rounding the number to an integer. Repeat participation was allowed in each group for a cross-over design. At first, all the participants were eighteen. In the crossover experiment, ten participants of eighteen dropped out for personal reasons. Of the final ten participants, ten participated in the 225 mg∙kg^−1^ enteric-coated tablet group and seven of them participated in the 225 mg∙kg^−1^ and 300 mg∙kg^−1^ uncoated tablet groups. Therefore, the compliance rate was 44% [(10 + 7 + 7)/(18 × 3) = (24/54) = 0.44]. As the crossover design was not fully conducted, along with poor compliance, statistical analysis was conducted through independent comparison, not through paired dependent comparison. The dosing interval was over three weeks so that the absorption of bicarbonate would not be affected by the administration in the previous participation. Half of the dose was administered with 150 mL of water (Evian^®^, Paris, France) within 5 min, and the other half was administered in the same manner after 30 min. Timing commenced at the administration of the first half dose. 

Before administration, intravenous catheters (BD Angiocath Plus, Franklin Lakes, NJ, USA) were inserted into all participants to take blood at each time point. Blood samples (1 mL) were drawn via the intravenous catheter into electrolyte-balanced heparinized syringes (BD Preset, Franklin Lakes, NJ, USA) every 30 min from 0 min (the actual time point was 10 min before the first half dose) to 360 min and analyzed immediately for bicarbonate (HCO_3_^−^), sodium (Na^+^), potassium (K^+^), chloride (Cl^−^), and lactate concentrations, as well as pH and pCO_2_ values, using an ABL 900 Flex Plus blood analyzer (Radiometer, Brønshøj, Denmark). 

Gastrointestinal symptoms including stomach bloating, belching, bowel urgency, stomachache, stomach cramps, flatulence, diarrhea, nausea, vomiting, and others were recorded through responses to a 10-item questionnaire. Symptoms were self-measured at each time point on a 10-point scale (no symptoms = 0 point, severe symptoms = 10 points). Symptom terminology was explained to participants before the experimental trial commenced to ensure consistency in the reporting of symptoms. All procedures in the experimental trial were performed and controlled by a nurse and a physician for the safety of the participants. 

### 2.5. Data Analysis

After the administration of sodium bicarbonate formulations, temporal profiles of various values, including bicarbonate concentration, were monitored for 360 min in the experimental human trial. In addition, changes in bicarbonate concentrations (ΔC) versus time were assessed to evaluate increases in blood bicarbonate concentrations by the formulations relative to the pre-existing endogenous bicarbonate. The duration time of bicarbonate in the blood was defined as the time needed to maintain a bicarbonate concentration above 5 mmol∙L^−1^, since performance improvement has been observed in most studies when the blood bicarbonate concentration increased by more than 5 mmol from the baseline [29]. This was calculated according to the change in bicarbonate concentration versus time.

Pharmacokinetic parameters were calculated using a non-compartmental analysis with a WinNonlin pharmacokinetic software (version 5.3) package (Pharsight, Mountain View, CA, USA). The area under the concentration versus time curve (AUC_0–t_) and the area under the change in bicarbonate concentration versus time curve (ΔAUC_0–t_) were calculated using the linear trapezoidal method. Peak blood bicarbonate concentration (C_max_), change in peak bicarbonate concentration (ΔC_max_), time to reach the peak (T_max_), mean residence time (MRT), and mean residence time in bicarbonate concentration change (ΔMRT) following administration were determined from the observed data. 

All data are expressed as mean and standard error of the mean (mean ± SEM). Normal Gaussian distribution of data was verified with the Kolmogorov–Smirnov test in the experimental human trial. With no normality found for any of the variables among groups (300 mg∙kg^−1^ uncoated tablets, 225 mg∙kg^−1^ uncoated tablets, and 225 mg∙kg^−1^ enteric-coated tablets), the non-parametric Kruskal–Wallis test was used with the Mann–Whitney U post hoc test for multiple comparisons (i.e., 300 mg∙kg^−1^ uncoated tablets vs. 225 mg∙kg^−1^ enteric-coated tablets and 225 mg∙kg^−1^ uncoated tablets vs. 225 mg∙kg^−1^ enteric-coated tablets) after adjusting the significance level by 0.05/2, according to the Bonferroni correction method. One-tail hypotheses were tested with a significance level set at *p* < 0.05. SPSS version 28.0 was used for all statistical analyses. Effect sizes were calculated using Hedge’s g for paired comparisons [41], and were described as small (g = 0.2), medium (g = 0.5), or large (g = 0.8) [42].

## 3. Results

### 3.1. Evaluation of Uncoated and Enteric-Coated Sodium Bicarbonate Tablets

The average weight, hardness, diameter, and thickness were 450.20 ± 14.52 mg, 42.36 ± 2.06 N, 9.52 ± 0.01 mm, and 4.45 ± 0.08 mm for uncoated tablets (*n* = 10) and 578.60 ± 8.72 mg, 237.32 ± 13.73 N, 9.92 ± 0.03 mm, and 5.15 ± 0.09 mm for enteric-coated tablets (*n* = 10), respectively. The friability was less than 1.0% for uncoated tablets (*n* = 20) and 0.05% for enteric-coated tablets (*n* = 20) after 4 min of tumbling. These values indicate that both tablets had good physical integrity. 

The disintegration time in pH 7.4 buffer was within 8 min for uncoated tablets (*n* = 6) and within 25 min for enteric-coated tablets (*n* = 6). The enteric-coated tablet surface was free from pores or cracks, indicating a consistent coating process. Enteric-coated tablets (*n* = 6) passed the disintegration test in pH 1.2 solution for 2 h, indicating that the enteric coating protected sodium bicarbonate under acidic conditions. 

### 3.2. Administration of Uncoated and Enteric-Coated Tablets in the Experimental Human Trial 

Blood samples were taken from participants before the experiment (10 min before taking the first half dose of tablets) to determine each participant’s blood bicarbonate concentration as each individual’s pre-experiment endogenous bicarbonate concentration in the blood. Sodium bicarbonate tablet formulations (i.e., uncoated tablets at a dose of 300 mg∙kg^−1^ sodium bicarbonate, uncoated tablets at a dose of 225 mg∙kg^−1^ sodium bicarbonate, and enteric-coated tablets at a dose of 225 mg∙kg^−1^ of sodium bicarbonate) were then administered in the experimental trial. 

**Figure 1:**
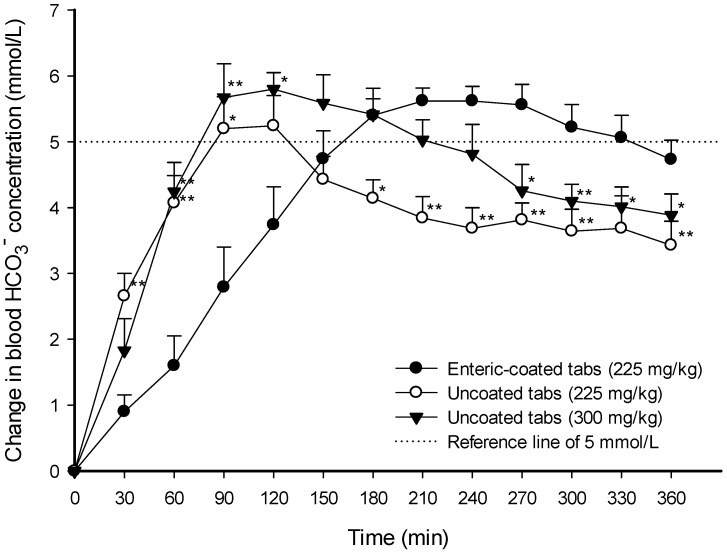
Average changes in blood bicarbonate concentrations versus time after oral administration of the following sodium bicarbonate formulations in an experimental human trial: (1) enteric-coated tablets equivalent to 225 mg∙kg^−1^ of sodium bicarbonate (●); (2) uncoated tablets equivalent to 225 mg∙kg^−1^ of sodium bicarbonate (○); and (3) uncoated tablets equivalent to 300 mg∙kg^−1^ of sodium bicarbonate (▼). Dotted line shows 5 mmol∙L^−1^, a reference considered sufficient to improve performance in high-intensity exercise. Each data point represents the mean ± standard error of the mean of 7–10 participants. *, *p* < 0.05 and **, *p* < 0.01 compared to the enteric-coated tablet formulation.

The mean (±SEM) changes in blood bicarbonate concentrations (ΔC) after administration of the formulations over time were analyzed to evaluate the increase in blood bicarbonate concentrations by the formulation compared to the pre-existing bicarbonate concentration (Figure 1). 

Figure 1 shows that the increase in the blood bicarbonate concentration changes was slower in the group given 225 mg∙kg^−1^ enteric-coated tablets than in the group given the uncoated tablets regardless of the dose, and the ΔC_max_ in the group given 225 mg∙kg^−1^ enteric-coated tablets, unlike in the group given 225 mg∙kg^−1^ uncoated tablets, was not lower than in the group given 300 mg∙kg^−1^ uncoated tablets. These bicarbonate absorption rates and concentration changes in each tablet formulation were analyzed through the pharmacokinetic parameters given in Table 1. 

The T_max_ for the group given 225 mg∙kg^−1^ enteric-coated tablets was increased by 1.85-fold (*p* < 0.01, g = 2.14) and 1.49-fold (*p* < 0.05, g = 1.16), respectively, compared to the groups given 225 mg∙kg^−1^ and 300 mg∙kg^−1^ uncoated tablets, although the group given 225 mg∙kg^−1^ enteric-coated tablets did not show statistically significant differences in Cmax or ΔC_max_ compared to both groups given uncoated tablets at doses of 225 mg∙kg^−1^ (C_max_: *p* = 0.81, g = 0.20; ΔC_max_: *p* = 0.13, g = 0.74) and 300 mg∙kg^−1^ (C_max_: *p* = 0.06, g = 1.10; ΔC_max_: *p* = 0.47, g = 0.29). In particular, the ΔAUC over Δ5 mmol∙L^−1^ and duration time over Δ5 mmol∙L^−1^ in the groups given 225 mg∙kg^−1^ enteric-coated tablets were significantly increased by 2.66-fold (*p* < 0.05, g = 0.72) and 2.48-fold (*p* < 0.05, g = 1.15) compared to the group given uncoated tablets at a dose of 225 mg∙kg^−1^, and were not statistically different from those given 300 mg∙kg^−1^ uncoated tablets (ΔAUC over Δ5 mmol∙L^−1^: *p* = 0.96, g = 0.04; duration time over Δ5 mmol∙L^−1^: *p* = 0.89, g = 0.06) (Table 1). Thus, the 225 mg∙kg^−1^ enteric-coated formulation increased T_max_ and showed no statistical differences in C_max_, ΔC_max_, ΔAUC over Δ5 mmol∙L^−1^, or duration time over Δ5 mmol∙L^−1^ compared to the 300 mg∙kg^−1^ uncoated formulation. 

In addition, the differences between the maximum and minimum values of pH, electrolytes (Na^+^, K^+^, Cl^−^), lactate, and pCO_2_ in blood for the group given 225 mg∙kg^−1^ enteric-coated tablets were not significantly different from those in the group given uncoated tablets at a dose of 225 or 300 mg∙kg^−1^, indicating that the enteric coating neither significantly reduced nor increased endogenous parameters (Table 2). 

### 3.3. Gastrointestinal Symptoms

Gastrointestinal symptoms such as stomach bloating, belching, bowel urgency, stomachache, stomach cramps, flatulence, diarrhea, nausea, and vomiting were self-measured, with 0 points indicating no symptoms and 10 points indicating severe symptoms at each blood sampling time point after the administration of each formulation. 

**Figure 2:**
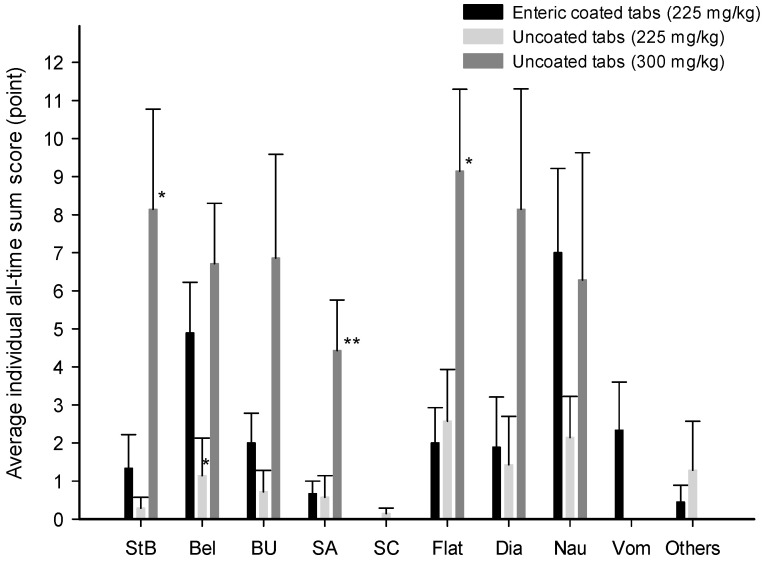
Average individual all-time sum scores for each symptom after oral administration of sodium bicarbonate tablet formulations in the experimental human trial. Each data point represents the mean ± standard error of the mean of 7–10 participants; *, *p* < 0.05 and **, *p* < 0.01 compared to the enteric-coated tablet formulation. (StB, stomach bloating; Bel, belching; BU, bowel urgency; SA, stomachache; SC, stomach cramps; Flat, flatulence; Dia, diarrhea; Nau, nausea; Vom, vomiting).

To compare the severity of each symptom according to the formulation, self-measured scores at each time point were summed individually for each symptom, and the average of the individual summed scores was compared for each formulation (Figure 2). The average of individual summed scores at all time points after administration of 225 mg∙kg^−1^ enteric-coated tablets did not significantly differ from that after administration of 300 mg∙kg^−1^ of uncoated tablets for symptoms of belching, bowel urgency, stomach cramps, diarrhea, nausea, vomiting, or others (belching, *p* = 0.47, g = 0.45; bowel urgency, *p* = 0.30, g = 0.99; stomach cramps, *p* = 1.00, g = 0.00 diarrhea, *p* = 0.14, g = 1.02; nausea, *p* = 0.84, g = 0.09; vomiting, *p* = 0.30, g = 0.79; others, *p* = 0.76, g = 0.43). In addition, the average of the individual summed scores with 225 mg∙kg^−1^ enteric-coated tablets did not significantly differ from those with 225 mg∙kg^−1^ uncoated tablets for any symptom except for belching (stomach bloating, *p* = 0.76, g = 0.50; bowel urgency, *p* = 0.30, g = 0.63; stomachache, *p* = 0.68, g = 0.08; stomach cramps, *p* = 0.68, g = 0.60; flatulence, *p* = 0.76, g = 0.18; diarrhea, *p* = 0.92, g = 0.12; nausea, *p* = 0.17, g = 0.89; vomiting, *p* = 0.30, g = 0.79; others, *p* = 0.92, g = 0.35). Interestingly, the stomach bloating, stomachache, and flatulence scores for participants given 225 mg∙kg^−1^ enteric-coated tablets were significantly lower by 0.16-fold (1.33 ± 0.88 vs. 8.14 ± 2.63, *p* < 0.05, g = 1.40), 0.15-fold (0.67 ± 0.33 vs. 4.43 ± 1.32, *p* < 0.01, g = 1.60), and 0.22-fold (2.00 ± 0.93 vs. 9.14 ± 2.15, *p* < 0.05, g = 1.70), respectively, than those for participants given 300 mg∙kg^−1^ uncoated tablets. On the contrary, the belching scores of participants given enteric-coated tablets increased by 4.28-fold compared to those of participants given uncoated tablets at a dose of 225 mg∙kg^−1^ (4.89 ± 1.34 vs. 1.14 ± 0.99, *p* < 0.05, g = 1.06). 

**Figure 3:**
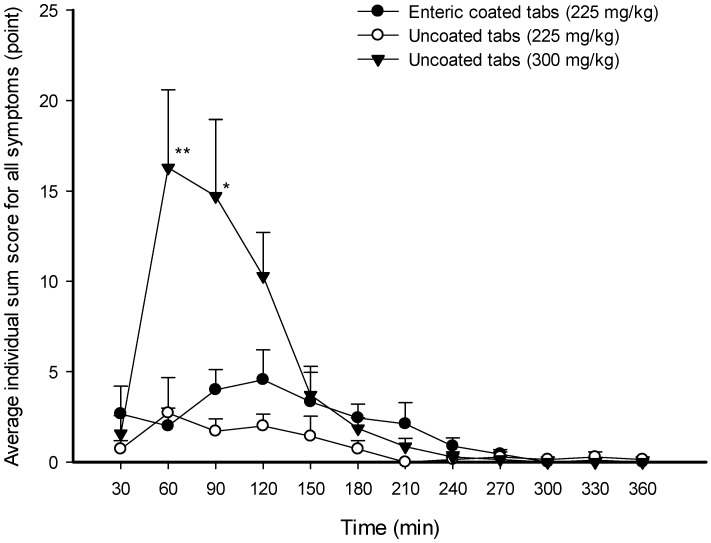
Average individual sum scores at each time point for all symptoms after oral administration of sodium bicarbonate tablet formulations in the experimental human trial. Each data point represents the mean ± standard error of the mean of 7–10 participants; * (*p* < 0.05) and ** (*p* < 0.01) compared to the enteric-coated tablet formulation.

To compare total symptom severity at each time point according to the formulation, the scores of all symptoms at each time point were summed individually, and the averages of the individual summed scores of all symptoms at each time point were compared for each formulation (Figure 3). The total symptom severity at each time point was not different between the group given enteric-coated tablets and the group given uncoated tablets at the same dose of 225 mg∙kg^−1^. However, at 60 min and 90 min, the total symptom scores of participants given 225 mg∙kg^−1^ of enteric-coated tablets were significantly decreased by 0.12-fold and 0.27-fold, respectively, compared to those given 300 mg∙kg^−1^ of uncoated tablets (2.00 ± 2.96 vs. 16.29 ± 11.40, *p* < 0.01 at 60 min, 4.00 ± 3.35 vs. 14.71 ± 11.22, *p* < 0.05 at 90 min) (Figure 3).

## 4. Discussion

The present study aims to evaluate the effects of intestinal delivery of sodium bicarbonate on bicarbonate absorption and associated side effects, and to confirm that enteric-coated bicarbonate tablets can reduce the dose. An experimental human trial was then conducted to evaluate the effects of intestinal delivery of sodium bicarbonate on pharmacokinetics and gastrointestinal symptoms.

Pharmacokinetic analysis of the experimental human trial showed that the change in absorption amount of bicarbonate over Δ5 mmol∙L^−1^ (ΔAUC over Δ5 mmol∙L^−1^) and the time to maintain a blood bicarbonate concentration change over 5 mmol∙L^−1^ (duration time over Δ5 mmol∙L^−1^) for the group given 225 mg∙kg^−1^ enteric-coated tablets were higher than those for the group given 225 mg∙kg^−1^ uncoated tablets, and did not differ statistically from those for the group given 300 mg∙kg^−1^ uncoated tablets. And the maximum blood bicarbonate concentrations (i.e., C_max_, ΔC_max_) after participants were given 225 mg∙kg^−1^ enteric-coated tablets were not statistically lower than those in participants given 300 mg∙kg^−1^ uncoated tablets. Moreover, the time to reach the maximum bicarbonate concentration (T_max_) for the group given enteric-coated tablets was increased compared to that for the group given 225 mg∙kg^−1^ or 300 mg∙kg^−1^ uncoated tablets. Also, the enteric-coated tablets resisted without porosity or cracking for 2 h at pH 1.2, and were fully dissolved at pH 7.4 in the disintegration test, indicating that the release of bicarbonate from the enteric-coated formulation occurred in the intestine. 

These results demonstrate that the extent of bicarbonate absorption over 5 mmol∙L^−1^ and the duration time at the lower dose were recovered by an enteric-coated formulation that extended the absorption time and protected sodium bicarbonate under acidic conditions. In addition, an individualized administration strategy is recommended, taking enteric-coated tablets before the time when 5 mmol/L is reached from the point at which exercise performance is needed. 

On the contrary, the finding of an increase in bicarbonate absorption in the group given the enteric-coated tablet formulation was not consistent with the results of previous studies. Hilton et al. reported that enteric-coated sodium bicarbonate capsules did not increase or change the blood bicarbonate concentration compared to uncoated capsules [5,36]. Although it is not clear, because they did not describe a disintegration test for enteric-coated capsules, it might be assumed that the difference in the effect of the enteric-coated formulation was due to the insufficient enteric coating or the material of the capsules rather than the difference between tablet and capsule formulations. 

Meanwhile, the delayed and extended absorption time (i.e., T_max_, duration time over Δ5 mmol∙L^−1^) and maintaining the extent of bicarbonate absorption (i.e., ΔAUC over Δ5 mmol∙L^−1^) without change in peak concentration (i.e., C_max_, ΔC_max_) might have contributed to decreases in undesirable gastrointestinal symptoms without changing pH, pCO_2_, lactate, or other electrolytes in participants given enteric-coated tablets. The severity scores for gastrointestinal symptoms, especially stomach bloating, belching, stomachache, and flatulence, were significantly decreased for the group given 225 mg∙kg^−1^ enteric-coated tablets, especially between 60 and 120 min, compared to those given 300 mg∙kg^−1^ uncoated tablets. 

The results, which stated that the common symptoms of 300 mg∙kg^−1^ uncoated tablets were flatulence, stomach bloating, diarrhea, and bowel urgency, were similar to the results of the previous study, which reported that the most common GI symptoms were stomach bloating, belching, and bowel urgency, and the symptoms with the highest severity were diarrhea, bowel urgency, and flatulence [36]. In addition, the reduced symptom severity in the group of enteric-coated formulations and the highest total symptom severity at 60 and 90 min in the group of 300 mg∙kg^−1^ uncoated tablets were consistent with the results of previous studies reporting that enteric-coated capsules resulted in fewer and less severe symptoms, and the highest incidence of gastrointestinal symptoms occurred at 60–90 min after administration of 300 mg∙kg^−1^ sodium bicarbonate gelatin or delayed-release capsule [36], as well as at 90 min after ingesting 300 mg∙kg^−1^ sodium bicarbonate solution [25].

Consequently, the enteric-coated formulations of 225 mg∙kg^−1^ tablets had an absorption capacity similar to that of 300 mg∙kg^−1^ uncoated tablets, and the effect of reducing the dose had the advantage of reducing the burden when taking a relatively small dose, as well as the effect of reducing side effects when taken by athletes. 

This study conducted a limited case study involving seven to ten participants to examine the effects of sodium bicarbonate. As a result, there was a limitation in that the verification power was kept low. However, as can be seen from the results of this study, significant differences appeared in the validation of essential differences, overcoming the limitation of low validation power and interpreting the results. Subsequent studies may require a randomized control trial (RCT) with sufficient case numbers to overcome the study’s limitation.

## 5. Conclusions

The present study suggests that the enteric-coated tablet formulation can reduce the oral dose of sodium bicarbonate by 25% from the most common oral dose of 300 mg·kg^−1^, which is required in order to achieve a sufficient blood bicarbonate concentration change to 225 mg·kg^−1^. It can also decrease gastrointestinal discomfort associated with the high oral dose. 

## Figures and Tables

**Table 1:** Pharmacokinetic parameters of blood bicarbonate after oral administration of sodium bicarbonate tablet formulations in an experimental human trial.

	AUC_0–360 min_ (min∙μmol∙mL^−1^)	C_max_ (μmol∙mL^−1^)	T_max_ (min)	ΔAUC over Δ5 mmol∙L^−1^ (min∙μmol∙mL^−1^)	ΔC_max_ (μmol∙mL^−1^)	Duration Time over Δ5 mmol∙L^−1^ (min)
Enteric-coated tabs (225 mg∙kg^−1^)	10,345 ± 110	31.0 ± 0.49	198.0 ± 16.9	118.5 ± 36.2	6.28 ± 0.26	143.3 ± 18.8
Uncoated tabs (225 mg∙kg^−1^)	10,420 ± 136 (g = 0.21)	30.6 ± 0.63 (g = 0.20)	107.1 ± 6.1 ** (g = 2.14)	44.6 ± 30.2 * (g = 0.72)	5.54 ± 0.46 (g = 0.74)	57.8 ± 34.8 * (g = 1.15)
Uncoated tabs (300 mg∙kg^−1^)	10,972 ± 161 ** (g = 1.64)	32.6 ± 0.53 (g = 1.10)	132.9 ± 22.5 * (g = 1.16)	123.2 ± 46.2 (g = 0.04)	6.53 ± 0.35 (g = 0.29)	147.2 ± 26.7 (g = 0.06)

Each data point represents the mean ± standard error of the mean of 7–10 participants; Hedge’s g, * (*p* < 0.05), and ** (*p* < 0.01) were compared to the enteric-coated tablet formulation (AUC_0–360min_, area under the concentration versus time curve; C_max_, peak bicarbonate concentration; T_max_, time to reach the peak bicarbonate concentration; ΔAUC over Δ5 mmol∙L^−1^, area under the change in bicarbonate concentration versus time curve where the change is over 5 mmol∙L^−1^; ΔC_max_, change in peak bicarbonate concentration; duration time over Δ5 mmol∙L^−1^, time to maintain a bicarbonate concentration change over 5 mmol∙L^−1^).

**Table 2:** Differences between the maximum and minimum values of pH (ΔpH), electrolytes, lactate, and pCO_2_ in blood after oral administration of sodium bicarbonate tablet formulations in an experimental human trial.

	ΔpH	Na^+^ (mmol∙L^−1^)	K^+^ (mmol∙L^−1^)	Cl^−^ (mmol∙L^−1^)	Lactate (mmol∙L^−1^)	pCO_2_ (mmHg)
Enteric-coated tabs (225 mg∙kg^−1^)	0.09 ± 0.01	5.20 ± 0.85	0.63 ± 0.11	3.80 ± 0.29	0.72 ± 0.14	35.09 ± 5.25
Uncoated tabs (225 mg∙kg^−1^)	0.09 ± 0.01 (*p* = 0.47, g = 0.17)	4.86 ± 0.77 (*p* = 0.81, g = 0.14)	0.74 ± 0.13 (*p* = 0.42, g = 0.33)	4.14 ± 0.63 (*p* = 0.36, g = 0.27)	0.43 ± 0.13 (*p* = 0.38, *g* = 0.74)	25.37 ± 2.69 (*p* = 0.13, g = 0.71)
Uncoated tabs (300 mg∙kg^−1^)	0.09 ± 0.01 (*p* = 0.81, g = 0.01)	4.43 ± 0.48 (*p* = 0.54, g = 0.34)	0.87 ± 0.18 (*p* = 0.31, g = 0.61)	4.29 ± 0.47 (*p* = 0.60, g = 0.46)	0.79 ± 0.13 (*p* = 0.45, g = 0.17)	36.00 ± 4.24 (*p* = 0.47, g = 0.06)

Each data point represents the mean ± standard error of the mean of 7–10 participants. *p* and Hedge’s g compared to the enteric-coated tablet formulation are displayed in parentheses.

## Data Availability

Data are contained within the article.
